# Effectiveness of bright light therapy in patients suffering from unipolar or bipolar depression; a naturalistic study

**DOI:** 10.1192/j.eurpsy.2025.1079

**Published:** 2025-08-26

**Authors:** M. A. Riedinger, G. E. van Son, N. J. van der Wee, E. J. Giltay, M. de Leeuw

**Affiliations:** 1 Psychiatry, Leiden University Medical Center; 2 Outpatient clinic for mental disability and psychiatry; 3 Department of Care and Quality, GGZ Rivierduinen, Leiden; 4 Public Health & Primary Care, Health Campus The Hague, The Hague; 5 Outpatient clinic for bipolar disorder, GGZ Rivierduinen, Leiden, Netherlands

## Abstract

**Introduction:**

Depressive disorders, both unipolar (MDD) and bipolar (BD), impact patients and society greatly. In bipolar depression and seasonal affective disorder the episodic nature and periodicity relate to changes in circadian rhythms. Bright light therapy (BLT) is thought to ameliorate symptoms of depression through its influence on circadian rhythms. Effectiveness of BLT has not been thoroughly established in real-world clinical samples.

**Objectives:**

To assess effectiveness of BLT in a real-world patients with BD or MDD and assess effect on individual symptoms of depression.

**Methods:**

For seventy-four patients with depression Inventory of Depressive Symptoms – Self Rated (IDS-SR) scores were available through Routine Outcome Monitoring (ROM) used in BLT in the outpatient clinic for mood disorders. Patients received one or two weeks of BLT as usual care. Patients suffering from MDD (n=33, 60.6% female, mean age 36.1±11.5 years) were compared to patients suffering from BD (n=41, 70.7% female, mean age 45.0±14.5 years) and changes in individual symptoms were analyzed for these two groups as well as the whole cohort.

**Results:**

IDS-SR scores decreased significantly in both groups of patients and did not differ in effect size between the groups. Explorative analyses of the effects on individual items of the IDS-SR showed that items related to core symptoms of depression such a as mood, concentration and energy level showed the largest improvements.

**Image:**

**Figure:**
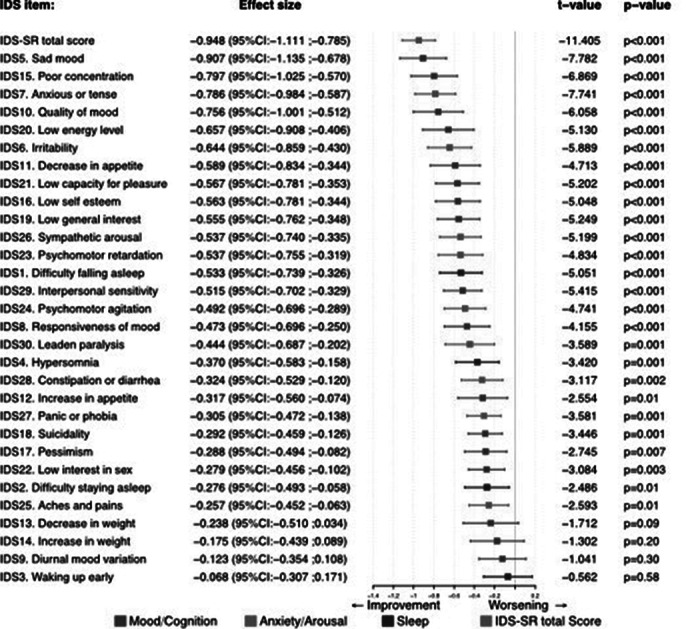

**Conclusions:**

Self-report depressive symptoms in patients suffering from either MDD or BD decreased in this naturalistic cohort after receiving BLT.

**Disclosure of Interest:**

None Declared

